# Hypoxia-inducible factor controls immunoregulatory properties of myeloid cells in mouse cardiac allografts – an experimental study

**DOI:** 10.1111/tri.13310

**Published:** 2018-07-13

**Authors:** Mikko A. I. Keränen, Alireza Raissadati, Antti I. Nykänen, Alexey Dashkevich, Raimo Tuuminen, Rainer Krebs, Randall S. Johnson, Simo O. Syrjälä, Karl B. Lemström

**Affiliations:** 1 Transplantation Laboratory, University of Helsinki, Helsinki, Finland; 2 Cardiac Surgery, Heart and Lung Center, Helsinki University Hospital, Helsinki, Finland; 3 Department of Physiology, Development & Neuroscience, University of Cambridge, Cambridge, UK; 4 Department of Cell and Molecular Biology, Karolinska Institute, Solna, Sweden

**Keywords:** differentiation, immune regulation, innate immunity, macrophage, maturation, monocyte biology

## Abstract

Hypoxia-inducible factors (HIFs) play a critical role in inflammatory properties of myeloid-derived cells. The effect of HIFs on myeloid-derived cell functions in organ transplantation remains unknown, however. We transplanted hearts into transgenic mice with myeloid cell-targeted deletions of HIF-1α or its negative regulator von Hippel–Lindau (VHL) to investigate the effects of HIF-1α inactivation or HIF pathway activation, respectively, on ischemia-reperfusion injury (IRI) and acute rejection. Deletion of VHL in myeloid cells enhanced mRNA expression of anti-inflammatory genes IDO, Arg-1, and HO-1 *in vitro. In vivo*, VHL^−/−^ myeloid-derived cells of allograft recipients alleviated IRI and acute rejection, evidenced by reduced cardiomyocyte damage, decreased proinflammatory cytokine mRNA levels, and absence of inflammatory infiltrate at 5 days after transplantation. Ultimately, allograft survival was significantly prolonged. *In vitro*, VHL^−/−^ myeloid-derived cells dose-dependently inhibited T-cell proliferation. Myeloid cells with HIF-1α-deletion retained proinflammatory qualities *in vitro* and *in vivo*. Deletion of VHL in myeloid cells of nonimmunosuppressed cardiac allograft recipients reduced myocardial injury and acute rejection. We suggest that HIF transcription factors induce a regulatory phenotype in myeloid-derived cells, which may be harnessed as a novel therapeutic strategy to regulate immune responses after heart transplantation.

## Introduction

The significance of ischemia-reperfusion injury and innate immunity in cardiac allograft rejection and survival is increasingly recognized [1]. Inflammatory cells of the myeloid lineage—including neutrophils, monocytes/macrophages, and dendritic cells (DC)—belong to the effector arm of the innate immune system. They promote the inflammatory response antigen-independently. Macrophages and DCs also serve as antigen-presenting cells, thus, bridging the innate and adaptive immune responses.

Hypoxia-inducible factors-1 and -2 (HIF-1 and HIF-2) are phylogenetically conserved αβ transcription factors that are composed of constantly synthesized, oxygen-regulated HIF-1α or HIF-2α subunits and a constitutively stable HIF-β subunit [2,3]. HIF-1α is widely expressed in all nucleated cells, whereas HIF-2α is restricted to some tissues [4]. In normoxia, HIF-α subunits are degraded by the von Hippel–Lindau (pVHL) protein, encoded by the tumor suppressor gene VHL. The loss of VHL leads to accumulation of both isoforms [5]. Renal cell carcinoma and von Hippel–Lindau disease are prime examples of aberrant VHL tumor suppressor gene activity, resulting in the abnormal expression of genes that control cell proliferation, metabolism, invasion, and angiogenesis [6].

The role of the HIF pathway in inflammation is complex. Myeloid lineage-targeted loss of HIF-1α dampens inflammation *in vitro* and *in vivo* [7]. Similarly, loss of HIF-2α compromises the inflammatory and migratory activity of macrophages [8]. However, myeloid-derived cells such as tumor-associated macrophages (TAMs) and myeloid-derived suppressor cells (MDSC) acquire anti-inflammatory properties through HIF-2α [9,10]. Myeloid-derived suppressor cells ameliorate allograft rejection by inhibiting T-effector cells and by inducing a T regulatory phenotype [11].

In this study, we investigated the role of HIFs in myeloid cells of fully MHC-mismatched cardiac allo-graft recipient mice. We used recipient mice with a myeloid lineage-targeted deletion of either HIF-1α or VHL that leads to constant HIF-1α inactivation or activation of HIF-1α and HIF-2α, respectively. Our novel findings demonstrate that loss of myeloid cell VHL favored an immunosuppressive myeloid cell phenotype that significantly reduced ischemia-reperfusion injury (IRI) and acute rejection, and ultimately prolonged cardiac allograft survival. Activation of HIF in myeloid cells may offer a novel therapeutic strategy for regulation of innate and, eventually, adaptive immune responses in an allogeneic environment.

## Methods

### Experimental design

We examined the inflammatory responses against fully MHC-mismatched cardiac allografts in recipient mice with myeloid lineage-targeted deletion of the hypoxia-responsive transcription factor HIF-1α, or its negative regulator VHL. C57Bl/6 mice carrying a floxed HIF-1α or VHL (loxP) allele were crossed with C57Bl/6 mice carrying the lysozyme M promoter (LysMCre) allele [12]. This resulted in specific deletion of the floxed gene in the myeloid lineage. The efficiency of CRE recombinase-mediated deletion in cells is 60–90% [7]. The mice were homozygously deficient in HIF-1α (resulting in constant HIF-1α inactivation) or pVHL (resulting in constant activation of HIF-1α and/or HIF-2α) in myeloid cells and are later referred to as mHIF-1α^−/−^ and mVHL^−/−^ mice, respectively. Wild-type littermates (LMs) with homozygously floxed either HIF or VHL, without Cre, served as controls.

First, we characterized the gene expression profile of myeloid-derived cells from mHIF-1α^−/−^, mVHL^−/−^, or LM mice *in vitro* (*n =* 6, 6, 6). Next, fully MHC-mismatched allogeneic heart transplantations were performed from specific pathogen-free Balb/C donor mice (Scanbur, Sollentuna, Sweden) to mHIF-1α^−/−^, mVHL^−/−^, or LM C57Bl/6-recipient mice to investigate the development of ischemia-reperfusion injury at 6 h after reperfusion (*n* = 6, 8, 15), acute rejection at 5 days after transplantation during moderate inflammation (*n* = 9, 5, 6), and allograft survival (*n* = 8, 5, 10). Allograft survival was defined as a palpable beat, without any special emphasis on beating rates. Additional syngeneic transplantations between wild-type fully MHC-matched Balb/C mice were performed as a control group for the ischemia-reperfusion model (*n* = 6). All operations were performed in a similar manner, and all heart transplants were comparably perfused. In the IRI-model, all heart transplants were subjected to 2 h of *ex vivo* cold preservation prior to transplantation into recipients. Allograft recipients did not receive any immunosuppression. Finally, we analyzed the anti-inflammatory properties of mVHL^−/−^ myeloid-derived cells in a T-cell proliferation assay *in vitro*.

Permission for animal experimentation was obtained from the State Provincial Office of Southern Finland. The animals received good care in compliance with the “Guide for the Care and Use of Laboratory Animals” (National Academy of Sciences, 2011; ISBN 978-0-3-0-15400-0).

### Isolation of mice resident peritoneal myeloid-derived cells

The resident peritoneal myeloid-derived cells were isolated as previously described [7]. Briefly, the mice were killed with cervical dislocation. The resident peritoneal myeloid-derived cells were isolated by injecting 10 ml sterile, ice-cold PBS (Ca^2+^ and Mg^2+^ free) into the peritoneal cavity, followed by gentle abdominal massage. The abdominal skin was incised through the linea alba to avert bleeding and the peritoneal fluid carefully collected. The cell suspension was centrifuged at 400 *g* at +4 °C for 10 min, washed once with PBS, dissolved in sterile water for 15 s to lyse any contaminating erythrocytes, and finally, the cell pellet was taken up in RPMI containing 10% heat-inactivated fetal bovine serum, penicillin 100 units/ml, and streptomycin 100 μg/ml. Cells were plated and allowed to adhere for 2–4 h. Non-adherent cells were washed off with PBS, and the adhered cells were collected for mRNA isolation and subsequent quantitative real-time RT-PCR analysis.

### Mouse heart transplantation

All animal procedures were performed with the mice under isoflurane anesthesia (2–5%/l O_2_). All mice received postoperative subcutaneous buprenorphine 0.15 mg/kg. We administered an intravenous bolus of ice-cold heparinized PBS to anesthetized donor mice to induce cardiac arrest and enhance cardioprotection during the subsequent surgery. Donors were killed immediately thereafter, and their hearts procured for transplantation. A midline incision was performed on the recipient, and the aorta and pulmonary artery of the allograft were anastomized to the recipient abdominal aorta and inferior vena cava, respectively. The anastomosis stage was completed in 1 h. At the end-point of the follow-up, recipients were killed and the heart transplant apex was stored in RNAlater, the mid-piece in Tissue-Tek and snap-frozen in liquid nitrogen and isopentane, and the base in paraformaldehyde.

### RNA isolation and quantitative real-time RT-PCR

Total RNA was extracted from peritoneal lavage cell lysates and from heart samples using RNeasy Mini Kit (Qiagen, Hilden, Germany) and reverse transcribed using the High-RNA-to-cDNA kit (Applied Biosystems, Foster City, CA, USA). qPCR analyses were carried out in a RotorGene-6000 (Corbett Research, Doncaster, Australia) using 2× Dynamo Flash SYBR Green Master mix (Finnzymes, Espoo, Finland). The number of mRNA copies of each gene of interest was calculated from a corresponding standard curve using the Rotor-Gene software (Corbett Research). The results are given in relation to 18S RNA expression.

### Immunohistochemistry

Cryostat sections were stained using the peroxidase ABC method (Vectastain Elite ABC Kit; Vector Laboratories, Burlingame, CA, USA), and the reactions were revealed by 3-amino-9-ethylcarbazole (AEC; Vector Laboratories). Antibodies and dilutions used were MPO for neutrophils (20 μg/ml, AB9535; Abcam, Cambridge, UK); CD4 (5 μg/ml, 22021D), and CD8 for T cells (5 μg/ml, 22071D), CD11b for macrophages (5 μg/ml 22451D), and CD11c for dendritic cells (5 μg/ml, 557394; BD Biosciences, San Diego, CA, USA). Specificity controls were performed with the same immunoglobulin concentration of species- and isotype-matched antibodies. The allograft-infiltrating inflammatory cells were counted from four random fields from each quadrant of the section (16 fields altogether) and are shown as the mean number of positive cells per mm^2^. Cells from each sample slide were quantitated by two independent observers (A.R and M.A.I.K) in a blinded fashion.

### Antigen-independent polyclonal T-cell proliferation assay

Unfractionated splenocytes (majority of T cells with accessory B cells, macrophages, and dendritic cells) were isolated from mVHL^−/−^ or littermate mice with mechanical homogenization. The cell suspension was gravity-filtered through a 70-μm nylon mesh (BD FalconTM, San Diego, CA, USA) to remove large debris. The cell suspension was centrifuged at 400 *g* at +4 °C for 10 min, dissolved in sterile water for 15 s to lyse any contaminating erythrocytes, and finally, the cell pellet was taken up in RMPI.

The cells were cultured at 2 × 10^5^ cells per well on 96-well plates in RPMI and stimulated with mouse CD3/CD28 T Cell Expander (Dynabeads^®^; Thermo Fisher Scientific, Waltham, MA, US) in 1:1 ratio. After 3 days of culture, the wells were pulsed with 3H-thymidine (3.7 × 10^4^ Bq per well; GE Healthcare, Bucking-hamshire, UK). The cells were harvested 6 h later with a Skatron harvester (Newington, NH, USA) and analyzed with a Microbeta liquid scintillation counter (Wallac, Turku, Finland) using OptiScint HiSafe scintillation fluid (PerkinElmer, Waltham, MA, USA). Background in nonstimulated wells was subtracted to obtain the adequate proliferative response.

In the subsequent experiment, the splenocytes (littermate) were mixed with syngeneic peritoneal myeloid-derived cells (littermate) at 1:1 and (mVHL^−/−^) at 1:1, 1:2, and 1:4 ratio. The total cell number was at 2 × 10^5^ cells per well. The cells were stimulated, cultured, and measured as in the previous experiment.

### Statistics

All data are expressed as mean ± SEM and analyzed using SPSS for Mac version 20.0 (SPSS Inc., Chicago, IL, USA). We used the nonparametric Kruskall–Wallis with Dunn’s test in multiple comparisons and the nonparametric Mann–Whitney *U*-test in pairwise comparison. For the survival analysis, Kaplan–Meier with log rank (Mantel-Cox) was applied. *P* < 0.05 was regarded as statistically significant.

## Results

### mVHL^−/−^ myeloid-derived cells upregulate vasculoprotective and anti-inflammatory genes

We collected naïve peritoneal myeloid-derived cells from mHIF-1α^−/−^, mVHL^−/−^, and LM mice and analyzed mRNA of selected genes involved in myeloid-derived cell functions and HIF-signaling.

**Figure 1:**
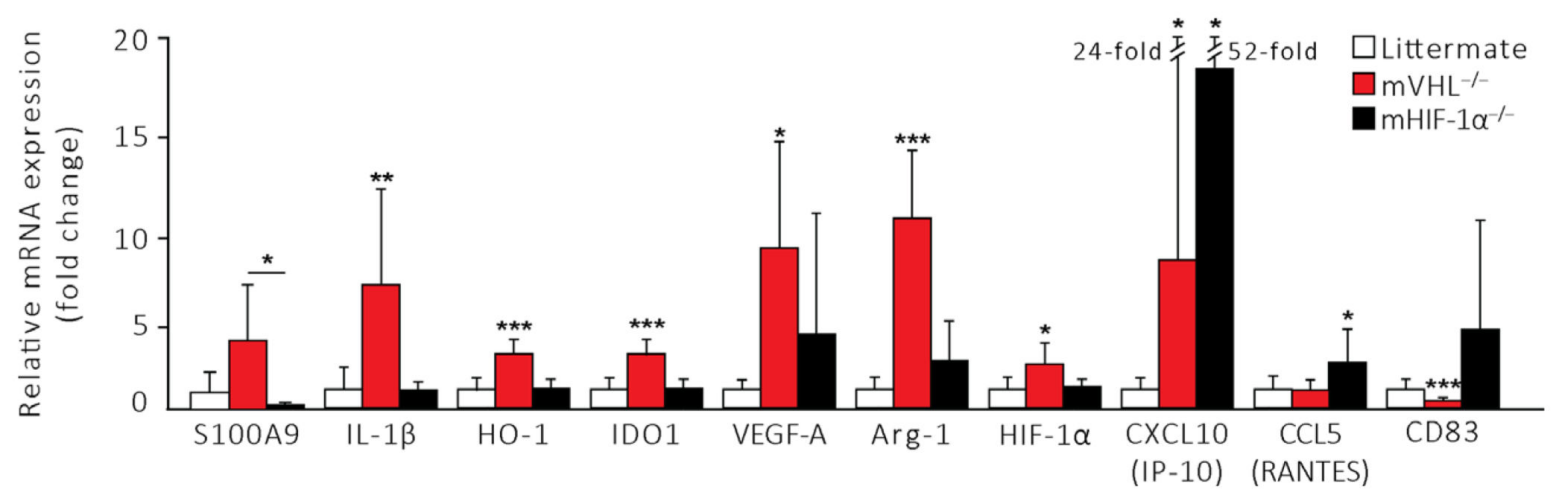
Deletion of VHL in myeloid-derived cells promotes a regulatory gene expression profile *in vitro*. Quantitative real-time RT-PCR analysis of mRNA expression profiles in resident peritoneal myeloid-derived cells from littermate, mHIF-1α^−/−^ or mVHL^−/−^ mice (*n =* 6, 6, 6). *P < 0.05, **P < 0.01, ***P < 0.005 by the Kruskal–Wallis test with Dunn’s test for multiple group and Mann–Whitney test for two-group comparisons.

mVHL^−/−^ myeloid-derived cells showed significantly increased transcription of IL-1β, VEGF-A, HO-1, IDO, and Arg-1 (Fig. 1, *P* < 0.05), which are all associated with MDSC functions [11,13,14]. Additionally, there was a trend toward increased expression of S100A9, an MDSC-associated factor (Fig. 1) [15]. However, we also observed increased mRNA levels of proinflammatory CXCL10 (IP-10) and VEGF-A in these same cells (Fig. 1, *P* < 0.05).

In contrast, HIF-1α inactivation in mHIF-1α^−/−^ macrophages resulted in upregulation of proinflammatory chemokines CXCL10 (IP-10) and CCL5 (RANTES) (Fig. 1, *P* < 0.05), and a trend toward increased expression of DC maturation marker CD83 (Fig. 1).

### Myocardial injury and proinflammatory cytokines are reduced in mVHL^−/−^ cardiac allograft recipients during ischemia-reperfusion injury

To evaluate the *in vivo* functional role of mHIF-1α^−/−^ and mVHL^−/−^ myeloid cells of cardiac allograft recipients, we performed heterotopic fully MHC-mis-matched heart transplantations into mHIF-1α^−/−^, mVHL^−/−^, or LM mice. To induce ischemic injury, donor hearts were subjected to 2 h of cold ischemic storage *ex vivo* prior to transplantation into recipients. Cardiac transplants were retrieved 6 h after reperfusion. There was no difference between the groups in the number of hearts beating at 6 h after reperfusion.

**Figure 2:**
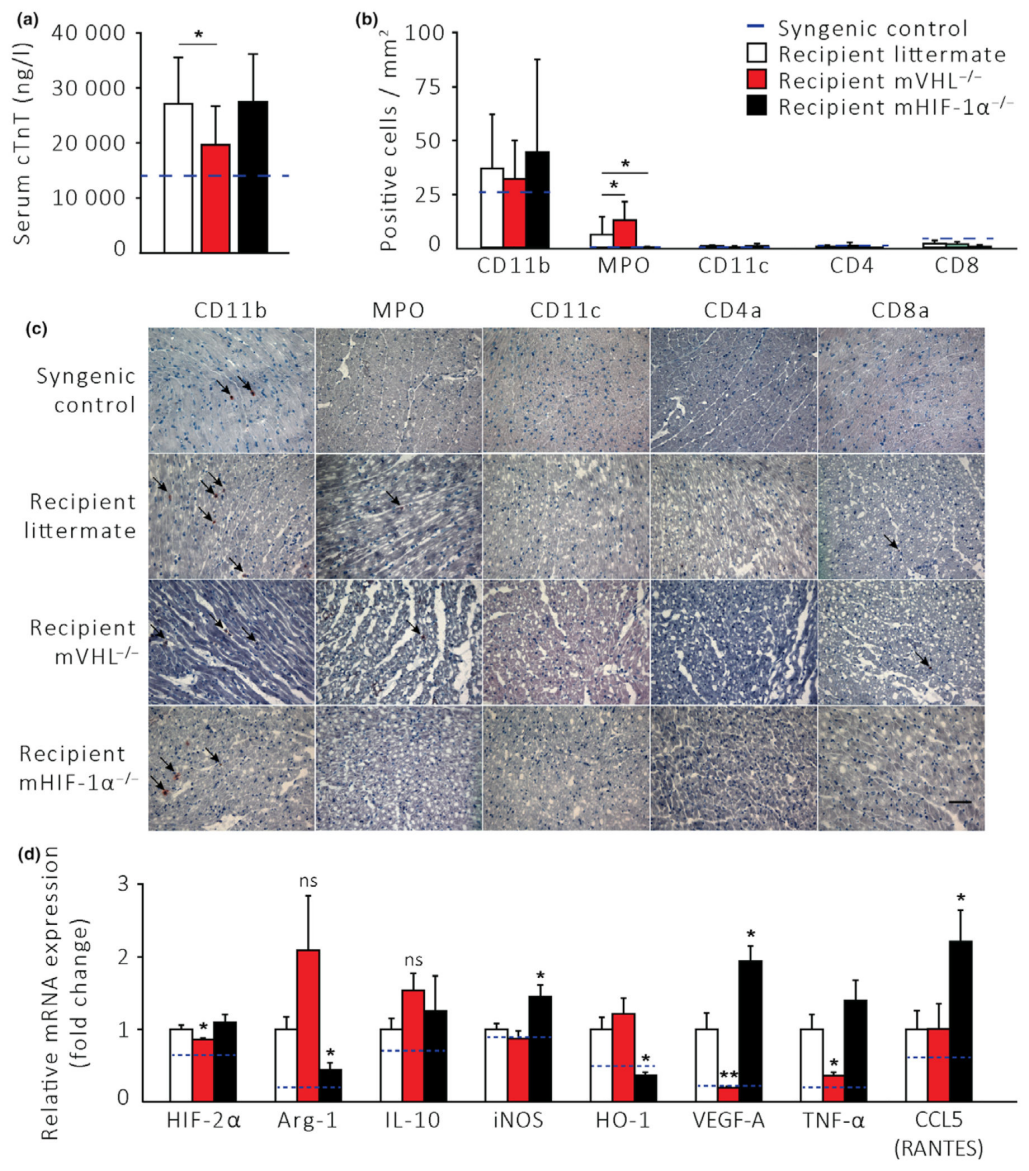
Ischemia-reperfusion injury is alleviated in fully MHC-mismatched cardiac allografts transplanted into mVHL^−/−^ recipient mice. Syngeneic (*n* = 6), littermate (*n* = 15), mVHL^−/−^ (*n* = 8), and mHIF^−/−^ (*n* = 6) mice were used as cardiac allograft recipients. (a) Serum release of cardiac troponin T (cTnT) in cardiac allograft recipients 6 h after reperfusion. (b) The number of allograft-infiltrating inflammatory cells 6 h after reperfusion. (c) Representative microphotographs of immunohistochemically stained samples. The scale-bar represents a distance of 5 μm. (d) The mRNA expression of inflammatory cytokines in cardiac allografts 6 h after reperfusion. *P < 0.05, **P < 0.01 by the Kruskal–Wallis test with Dunn’s test for multiple group and Mann–Whitney test for two-group comparisons.

In mVHL^−/−^ cardiac allograft recipients, the serum cardiac troponin T release as a biomarker for myocardial injury was significantly reduced at 6 h after reperfusion compared to LM recipients (Fig. 2a, *P* < 0.05). Analyses of frozen allograft cross-sections by immunohistochemistry revealed a comparable number of graft-infiltrating CD11b^+^ myeloid-derived cells, but a significantly higher number of intragraft MPO^+^ leukocytes in mVHL^−/−^ recipients 6 h after reperfusion (Fig. 2b and c, *P* < 0.05). Quantitative real-time RT-PCR from cardiac allografts revealed a significant decrease in mRNA expression of proinflammatory VEGF-A and TNF-α in mVHL^−/−^ recipients at 6 h after reperfusion compared to LMs (Fig. 2d, *P* < 0.01 and *P* < 0.05, respectively). Also, we observed a 1.5-fold and 2.1-fold increase in mRNA expression of anti-inflammatory Il-10 and Arg-1, respectively, in cardiac allografts transplanted into mVHL^−/−^ mice compared to LMs, although no statistical significance was observed (Fig. 2d, nonsignificant). Interestingly, the mRNA level of HIF-2α was significantly reduced in mVHL^−/−^ cardiac allografts compared to LM mice (Fig. 2d, *P* < 0.05).

In contrast, in mHIF-1α^−/−^ cardiac allograft recipients, the degree of myocardial injury and the infiltration of inflammatory cells into allografts remained unchanged compared to LMs (Fig. 2a–c). However, cardiac allograft mRNA levels of proinflammatory iNOS, VEGF-A, and CCL5 (RANTES) were increased in mHIF-1α^−/−^ recipients, with a corresponding decrease in anti-inflammatory Arg-1 and HO-1 mRNA levels at 6 h after reperfusion (Fig. 2d, *P* < 0.05).

There was no difference in the mRNA expression levels of IL-1β, IL-6, IL-12p35, CXCL10 (IP-10), CCL2 (MCP-1), CCL3 (MIP-1α), CCL5 (RANTES), IDO1, HIF-1α, Foxp3, STAT3, CD83, or CD80 between the groups.

### Acute rejection is diminished in cardiac allografts transplanted into mVHL^−/−^ recipients

To determine the role of myeloid cell HIF pathway in the development of allograft rejection, we performed cardiac transplantations from fully MHC-mismatched wild-type Balb/C donors to nonimmunosuppressed mHIF-1α^−/−^, mVHL^−/−^, or LM C57 mice without additional *ex vivo* cold preservation. We retrieved the allografts on day 5 after transplantation and analyzed histology, the degree of intragraft leukocyte infiltration, and the cytokine and chemokine mRNA expression profile.

**Figure 3:**
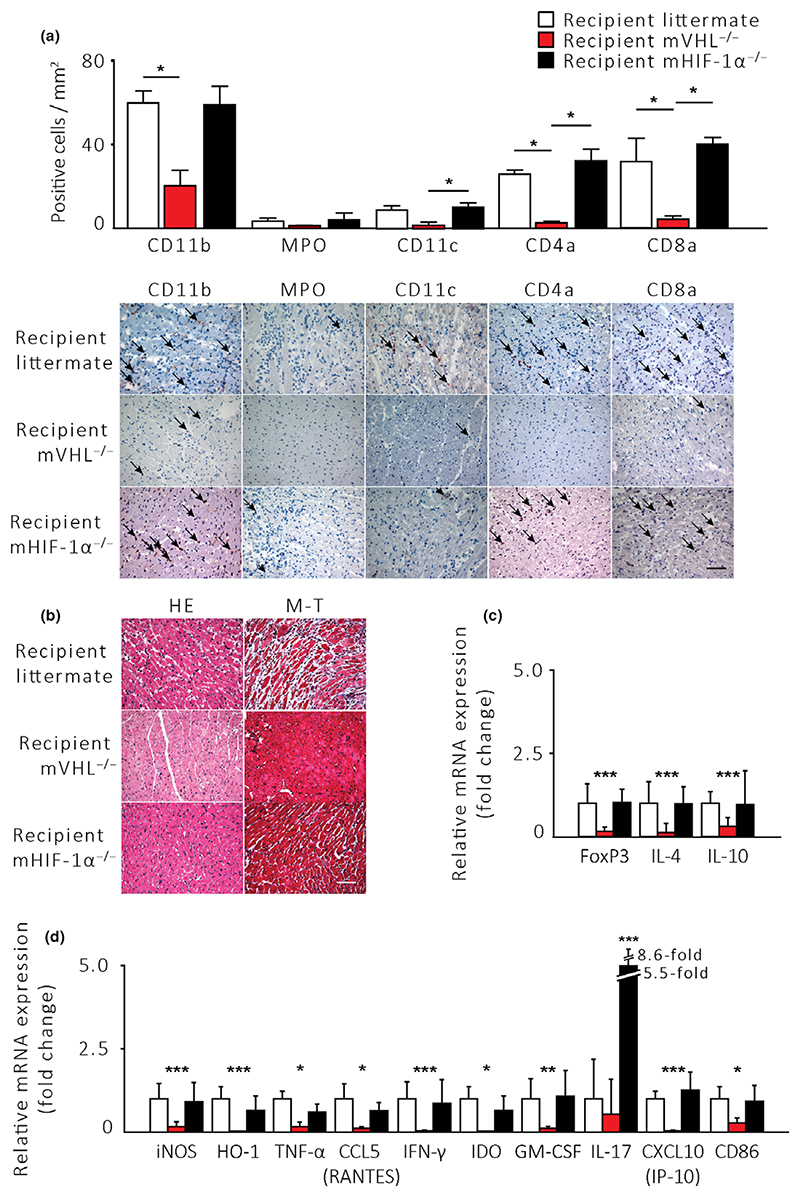
Acute rejection is reduced in fully MHC-mismatched cardiac allografts transplanted into mVHL^−/−^ recipients. Littermate (*n* = 6), mVHL^−/−^ (*n* = 5), and mHIF^−/−^ (*n* = 9) mice were used as cardiac allograft recipients. (a) Number of cardiac allograft-infiltrating inflammatory cells 5 days after transplantation with representative microphotographs of immunohistochemically stained samples. (b) Microphotographs 5 days after transplantation show well-preserved myocardium in hematoxylin and eosin and Masson’s trichrome stains. mRNA expression of (c) anti-inflammatory and (d) inflammatory cytokines in cardiac allografts 6 h after reperfusion. The scale-bar represents a distance of 5 μm. *P < 0.05, **P < 0.01, ***P < 0.005 by the Kruskal–Wallis test with Dunn’s test for multiple group and Mann–Whitney test for two-group comparisons.

In cardiac allografts transplanted into mVHL^−/−^ recipients, the number of graft-infiltrating CD11b^+^ myeloid-derived cells, CD11c^+^ dendritic cells, and CD4^+^ and CD8^+^ T cells was reduced compared to allografts transplanted into LM recipients (Fig. 3a, *P* < 0.05). We observed no difference in the number of MPO^+^ leukocytes between the groups (Fig. 3a). Minuscule inflammatory cell infiltration and well-preserved myocardium (tissue architecture) in mVHL^−/−^ recipients were also evident from the general inspection of the immunohistochemically and histologically stained sections (Fig. 3a and b).

Furthermore, in mVHL^−/−^-recipient mice, the allograft mRNA expression of proinflammatory factors TNF-α, IFN-γ, IL-4, iNOS, CXCL10 (IP-10), CCL5 (RANTES), and GM-CSF was decreased (Fig. 3d, *P* < 0.05). We also observed a significant decrease in antigen-presenting cell maturation marker CD86 in allografts transplanted into mVHL^−/−^ mice. Interestingly, cardiac allograft expression levels of anti-inflammatory factors IL-10, FoxP3, IDO, and HO-1 were also significantly reduced in mVHL^−/−^ recipients compared to LM recipients (Fig. 3c).

Loss of HIF-1α in the recipient myeloid cells did not affect the allograft inflammatory cell influx but rather caused a significant upregulation of proinflammatory IL-17 compared to LMs (Fig. 3d, *P* < 0.05).

### Cardiac allograft survival is significantly prolonged in mVHL^−/−^ recipients

The results at 5 days after transplantation suggested a blunted cardiac allograft acute rejection and adaptive immune response in mVHL^−/−^ recipients. We investigated the long-term implications of this finding by analyzing the survival of cardiac allografts transplanted into nonimmunosuppressed mHIF-1α^−/−^, mVHL^−/−^, or LM recipients. Allograft survival was monitored by daily abdominal palpation of the heartbeat, and mRNA expression and inflammatory cell infiltration were analyzed at the end of the follow-up.

**Figure 4:**
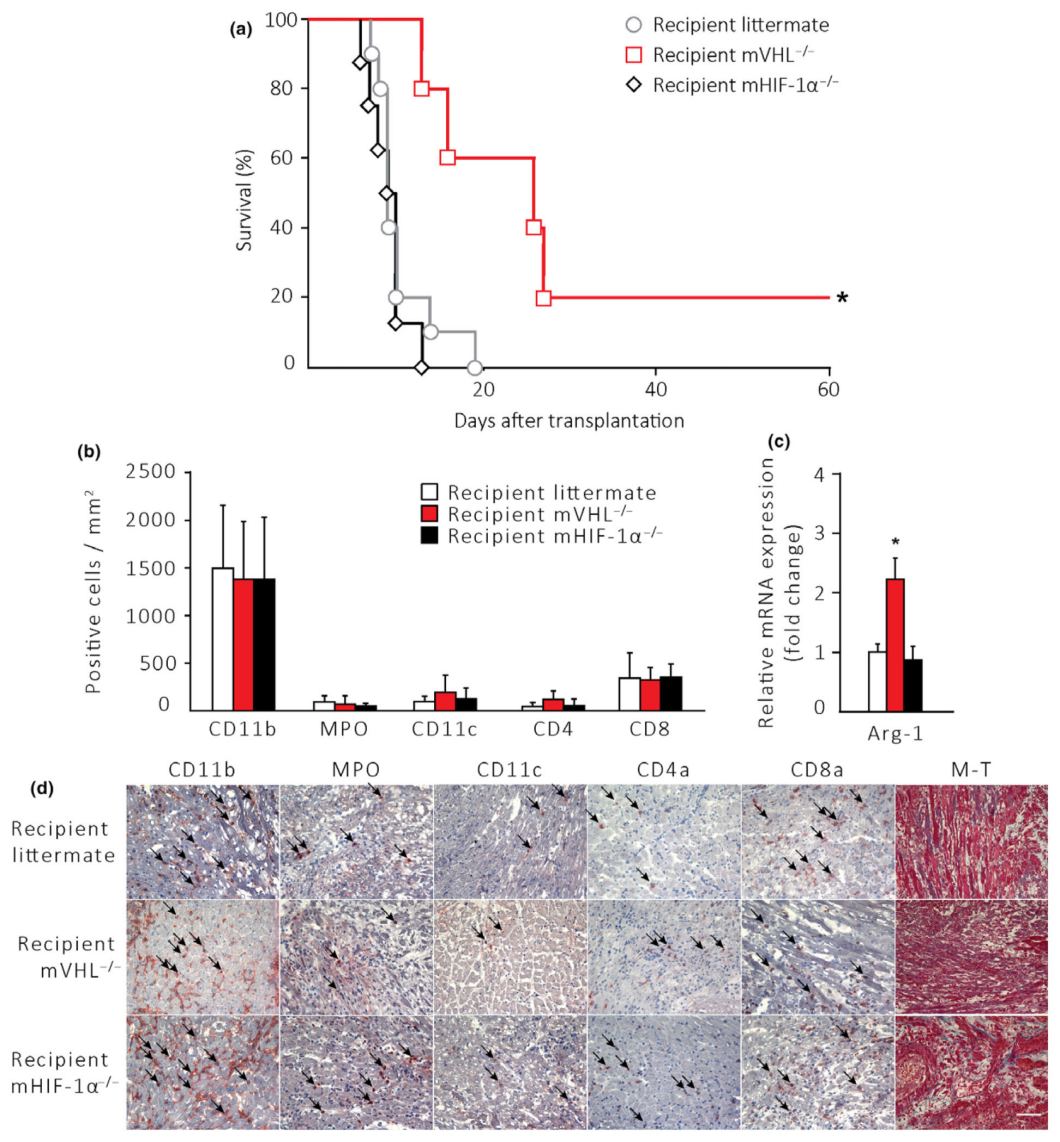
Cardiac allograft survival is prolonged in mVHL^−/−^ recipients. Littermate (*n* = 10), mVHL^−/−^ (*n* = 5), and mHIF^−/−^ (*n* = 8) mice were used as cardiac allograft recipients. (a) Allograft survival was measured by daily abdominal palpation of contractile activity of the transplant. *P < 0.05 by log-rank test. (b) The number of allograft-infiltrating leukocytes at the time of graft failure. (c) Allograft Arg-1 mRNA expression. (d) Representative microphotographs of immunohistochemically stained samples. The scale bar represents a distance of 5 μm. *P < 0.05 by log-rank test for survival statistics and by the Kruskal–Wallis test with Dunn’s test for multiple group and Mann–Whitney test for two-group comparisons of means.

The median survival of allografts transplanted to LM and mHIF-1α^−/−^ recipients was similar with 10 ± 1 and 9 ± 1 days, respectively. The median survival of allografts transplanted into mVHL^−/−^ recipients was significantly prolonged to 20 ± 4 days, (Fig. 4a, *P* < 0.05). The number of CD11b^+^, MPO^+^, or CD11c^+^ myeloid-derived cells and CD4^+^ and CD8^+^ T cells was similar between the groups (Fig. 4b). We observed, however, a significantly increased anti-inflammatory Arg-1 mRNA expression in whole-tissue samples from hearts transplanted into mVHL^−/−^ mice at the end-point of the survival model (Fig. 4c). There were no differences in the mRNA expression of IL-10, iNOS, HO-1, IFN-γ, VEGF-A, CCL-3 (MIP-1α), IL-6, IL-33, or CCL5 (RANTES) at the time of graft failure (data not shown).

### mVHL^−/−^ myeloid-derived cells suppress lymphocyte proliferation *in vitro*

The adaptive immune response was significantly delayed in allografts transplanted into mVHL^−/−^ mice compared to LM recipients. To further investigate this immunoregulatory effect of HIF pathway activation in myeloid-derived cells, we analyzed *in vitro* whether mVHL^−/−^ myeloid-derived cells were capable of suppressing T-cell proliferation in an antigen-nonspecific proliferation assay.

**Figure 5:**
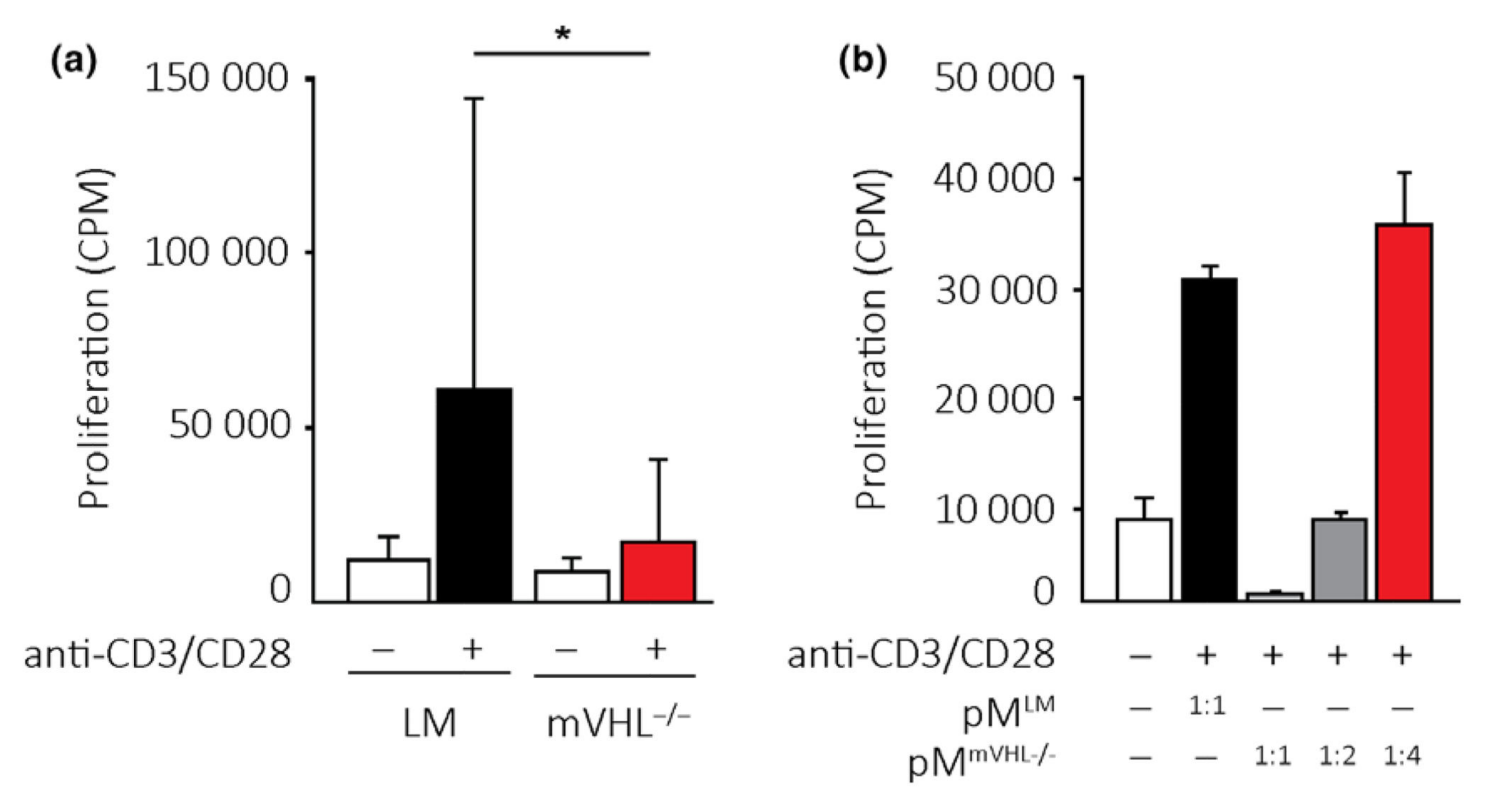
mVHL^−/−^ myeloid-derived cells inhibit T-cell proliferation *in vitro*. (a) Unprimed splenocytes from littermate (LM) or mVHL^−/−^ mice were stimulated with anti-CD3/CD28 antibodies. (b) Littermate or mVHL^−/−^ myeloid-derived cells were co-cultured at indicated ratios with unprimed splenocytes from littermate mice (no allogeneic setting) and stimulated with CD3/CD28 antibodies. *P < 0.05 by the Kruskal–Wallis with Dunn’s test for multiple group.

First, unfractionated and unprimed splenocytes including T cells and myeloid accessory cells were stimulated with mouse CD3/CD28 antibodies. Stimulation of splenocytes from LM mice induced a significant proliferation response, whereas stimulation of mVHL^−/−^ splenocytes resulted in significantly reduced proliferation response of these cells (Fig. 5a, *P* < 0.05).

Next, the unfractionated and unprimed splenocytes from the LM were stimulated with mouse CD3/CD28 antibodies and co-cultured with peritoneal myeloid-derived cells either from LM or mVHL^−/−^ mice. Co-culture of splenocytes with mVHL^−/−^ peritoneal myeloid-derived cells inhibited splenocyte proliferation in a dose-dependent manner (Fig. 5b).

## Discussion

We demonstrate that the HIF pathway in myeloid-derived cells may induce a regulatory phenotype. These immunoregulatory cells were able to migrate into cardiac allografts within 6 h after reperfusion and suppress the early innate inflammatory response and allograft damage possibly with increased expression of IL-10 and Arg-1 and reduced the expression of proinflammatory VEGF-A and TNFα. They thereby significantly dampened the ensuing adaptive immune response and acute rejection, as evidenced by minuscule inflammatory cell infiltrate, well-preserved tissue architecture, significantly reduced expression of proinflammatory cytokines and chemokines on the 5th postoperative day, and a subsequently prolonged allograft survival. We also demonstrate the capability of these HIF-activated myeloid-derived cells to directly suppress the proliferation of CD3/CD28 antibody-activated splenocytes *in vitro*. Finally, we provide evidence that myeloid-derived cells retained their inflammatory properties despite the loss of HIF-1α and effectively migrated to and rejected cardiac allografts.

Regulatory myeloid-derived cells are an expanding subset of leukocytes including MDSCs, alternatively activated (M2) macrophages, regulatory macrophages, tumor-associated macrophages (TAM), tolerogenic DCs, and regulatory neutrophils (N2) [10,16,17]. The significant overlap of cell surface markers and gene expression profiles renders their characterization challenging, however. Myeloid-derived suppressor cells are characterized by STAT3 and S100A8/9 expression, M2 macrophages by STAT6- and Arg-1 expression, and regulatory macro-phages by lack of STAT6 but high IL-10 expression [15,17–19]. Tolerogenic DCs, on the other hand, express high levels of indoleamine-2,3-dioxygenase (IDO) [20]. Nevertheless, factors such as Arg-1, IL-10, TGF-β, HO-1 are all expressed by MDSCs, M2 macrophages, TAMs, and regulatory macrophages alike [10,16–19]. In this study, the majority of these factors were also highly expressed by the mVHL^−/−^ myeloid-derived cells *in vitro*, suggesting that mVHL^−/−^ results in a heterogeneous mix of myeloid cells with various potentially immunosuppressive properties.

The role of hypoxia and HIFs in inflammatory processes remains ambiguous. In DC, hypoxia and HIFs may mediate both pro- and anti-inflammatory properties [21,22]. In macrophages, on the other hand, HIF-1α promotes a proinflammatory phenotype during LPS-induced sepsis, whereas HIF-2α drives the development and function of immunosuppressive TAMs that inhibit T-cell activity [9,23]. Furthermore, in a hypoxic tumor microenvironment, MDSCs acquire profound immunosuppressive qualities via HIF-1α-stabilization [24]. Conversely, lack of HIF-1α in myeloid cells slows tumor progression, indicating enhanced anti-tumor immunity [25]. Myeloid-derived suppressor cells normally reside in the hypoxic environment of the bone marrow with HIF-1α playing an important role in their development [26]. Thus, we suggest that constantly stable HIFs allow the mVHL^−/−^ myeloid-derived cells to retain their immunoregulatory properties outside their natural niche of hypoxic bone marrow.

We demonstrate that mVHL^−/−^ myeloid-derived cells suppressed splenocytes *in vitro*. Graft-infiltrating MDSCs are vital for induction of tolerance in anti-CD28 antibody-treated rat kidney transplant- and CD40 antagonist-treated mouse cardiac allograft recipients [27,28]. In our model, although the immunoregulatory effect of mVHL^−/−^ leukocytes prolonged graft survival, it merely delayed the adaptive immune response. Garcia *et al*. demonstrated that the first wave of tolerance-inducing allograft-infiltrating CD11b^+^CD115^+^GR1^+^ regulatory monocytes originated from the circulation but necessitated further chemokine- and growth factor-driven mobilization from the bone marrow to maintain their suppressive effect [28]. In mVHL^−/−^ recipients, we observed a decreased number of allograft-infiltrating CD11b^+^ myeloid-derived cells along with diminished expression of chemokines CCL5 and CXCL10, growth factor GM-CSF, and anti-inflammatory IL-10 and HO-1 at 5 days after transplantation. These findings may suggest that despite the initial CD11b^+^ regulatory cell influx into the allograft, the subsequent failure to mobilize and attract immunoregulatory cells from the recipient bone marrow to the allograft tipped the balance toward inflammation and eventual rejection of the allograft. As such, achieving sufficient and prolonged allograft immunoregulation with these mVHL^−/−^ myeloid-derived cells would most likely require successive adoptive transfers.

The important role of HIF-1α in myeloid cell-mediated inflammation has been previously established both *in vivo* and *in vitro* [7]. We previously found that pharmacologic stabilization of HIF in the heart donor exacerbated IRI [29]. Interestingly, in this study, we found that loss of HIF-1α in myeloid cells did not affect the influx of CD11b^+^ macrophages and CD11c^+^ dendritic cells into fully MHC-mismatched cardiac allografts at 6 h and 5 days after transplantation. Also, these leukocytes were fully capable of mounting an effective acute rejection, as evidenced by the comparable expression of proinflammatory factors between the mHIF-1α^−/−^ and LMs, and even higher IL-17 mRNA expression, but no difference in survival compared to LM mice. We obtained similar results in an experimental lung allograft model of mice in our previous study [30]. The complex role of HIFs in myeloid cell-mediated inflammation was extensively reviewed recently by Lin *et al*. [31] Multiple inflammatory models have concluded a seemingly ambiguous role of HIFs in various inflammatory conditions. In this study, HIFs mediated a protective function in the specific context of organ transplant recipient immunity. The contradicting results with our previous study may be explained by the whole-tissue-targeted HIF activation, including vascular endothelial cells and cardiomyocytes, in the 2013 study, versus the myeloid-derived cell-specific loss and activation of HIF in the current study [30].

### Study limitations

This experimental study aimed to investigate the biological effects of the HIF pathway of myeloid-derived cells on cardiac allograft rejection. The murine model, although excellent for experimental studies, does not translate directly into the clinical scenario. Also, the current model excluded T-cell immunosuppression. For future investigations, however, the potential effect of immunosuppressive medication on the regulatory effect of mVHL^−/−^ myeloid-derived cells should be considered. Calcineurin inhibitors target adaptive immune cells, which could potentiate the regulatory effect of mVHL^−/−^ myeloid-derived cells. Glucocorticoids, on the other hand, affect all immune cells, but have been shown to induce macrophage M2 differentiation [32].

Other direct downstream targets of pVHL have not been considered in this study. One notable factor is Akt, which promotes tumor cell survival [33].

## Conclusion

In summary, loss of VHL in myeloid-derived cells of cardiac allograft recipients reduced ischemia-reperfusion injury, acute rejection, and prolonged allograft survival. We postulate that mVHL^−/−^ myeloid-derived cells retained their immature, subsequently immunosuppressive, phenotype outside the bone marrow. The exact cellular phenotype of mVHL^−/−^ myeloid-derived cells was difficult to characterize but resembled that of MDSCs and M2 macrophages based on their surface markers and gene expression profile. Finally, we demonstrated that myeloid cell HIF-1α was not required for mounting an adaptive alloimmune response. Thus, our results suggest that genetic or pharmacological HIF activation in myeloid cells may be a novel therapeutic strategy to regulate innate and, therefore, adaptive immune responses after solid organ transplantation.

## Figures and Tables

**Figure 6:**
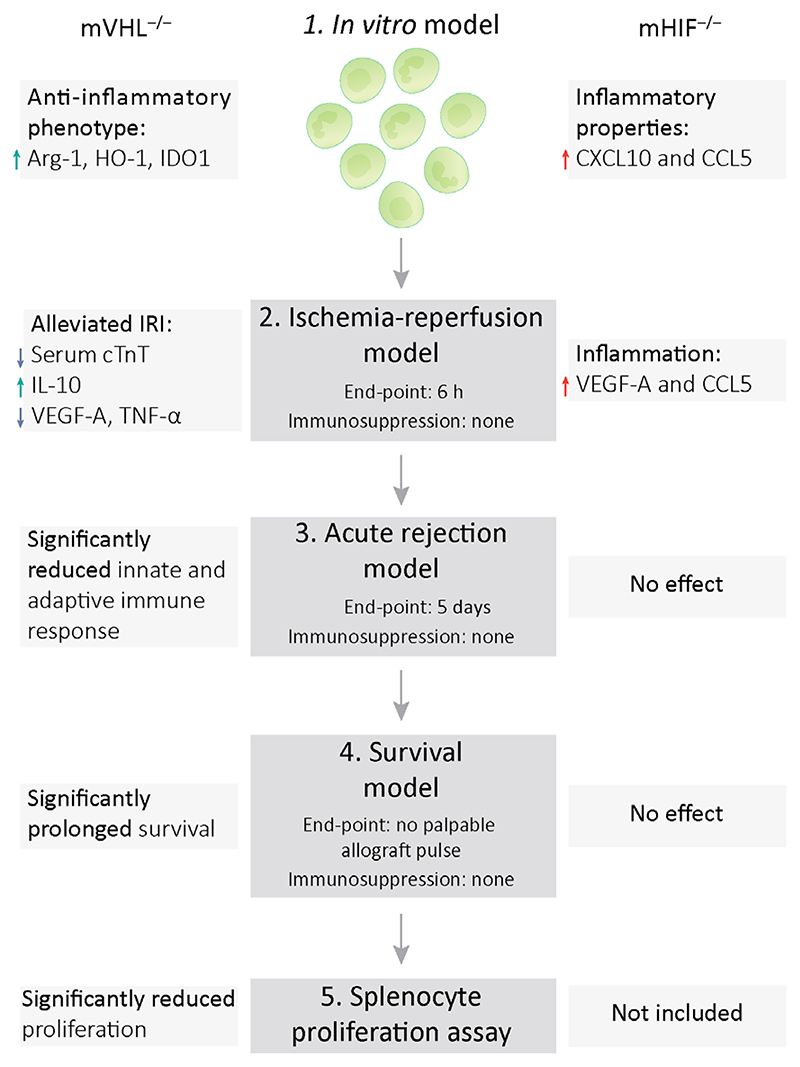
Pathway diagram of the study.
